# De novo truncating variant in NSD2gene leading to atypical Wolf-Hirschhorn syndrome phenotype

**DOI:** 10.1186/s12881-019-0863-2

**Published:** 2019-08-05

**Authors:** Yanrui Jiang, Huizhen Sun, Qingmin Lin, Zengge Wang, Guanghai Wang, Jian Wang, Fan Jiang, Ruen Yao

**Affiliations:** 1 Department of Developmental and Behavioral Pediatrics, Shanghai Children’s Medical Center, Shanghai Jiao Tong University School of Medicine, 1678 Dongfang Road, Shanghai, 200127 People’s Republic of China; 2 MOE-Shanghai Key Laboratory of Children’s Environmental Health, Shanghai, 200127 People’s Republic of China; 3 Department of Urology, Shanghai Children’s Hospital, Shanghai Jiao Tong University School of Medicine, Shanghai, 200127 People’s Republic of China; 4 Department of Medical Genetics and Molecular Diagnostic Laboratory, Shanghai Children’s Medical Center, Shanghai Jiao Tong University School of Medicine, 1678 Dongfang Road, Shanghai, 200127 People’s Republic of China

**Keywords:** Wolf-Hirschhorn syndrome, *NSD2* gene, Truncating variants

## Abstract

**Background:**

Wolf-Hirschhorn syndrome (WHS) is a contiguous gene syndrome caused by partial 4p deletion highly variable in size in individual patients. The core WHS phenotype is defined by the association of growth delay, typical facial characteristics, intellectual disability and seizures. The WHS critical region (WHSCR) has been narrowed down and *NSD2* falls within this 200 kb region. Only four patients with *NSD2* variants have been documented with phenotypic features in detail.

**Case presentation:**

Herein, we report the case of a 12-year-old boy with developmental delay. He had dysmorphic facial features including wide-spaced eyes, prominent nasal bridge continuing to forehead, abnormal teething and micrognathia. He also had mild clinodactyly of both hands. Using whole-exome sequencing, we identified a pathogenic mutation in *NSD2* [c.4029_4030insAA, p.Glu1344Lysfs*49] isolated from peripheral blood DNA. Sanger confirmation of this variant revealed it as a de novo truncating variant in the family.

**Conclusion:**

Here, we reported a boy with de novo truncating variant in *NSD2* with atypical clinical features comparing with 4p16.3 deletion related WHS. Our finding further supported the pathogenesis of truncating variants in *NSD2* and delineated the possible symptom spectrum caused by these variants.

## Background

Wolf-Hirschhorn syndrome (WHS) was first described in 1965 as a congenital anomalies/mental retardation due to partial deletion on p-terminal of chromosome 4 [1]. WHS (OMIM 194190) patients were characterized with craniofacial features including microcephaly, ‘Greek warrior helmet’ appearance of wide nasal bridge, widely spaced and prominent eyes, and distinct mouth with down-turned corners, short philtrum and micrognathia [2]. Other frequent features observed in WHS patients were intrauterine growth retardation, postnatal growth deficiency, intellectual disability, hypotonia, seizures, feeding difficulties and muscle hypotrophy [3]. Depending mostly on the extent of the 4p deletion, additional clinical signs include major malformations, as midline defects, congenital heart defects, renal and skeletal anomalies [4].

Overlapping regions of multiple cases diagnosed with WHS has helped to decide the critical region of WHS, namely WHSCR1 and WHSCR2, which has been narrowed down to a 200 kb region on 4p16.3 [5, 6]. Typical WHS, even in the mild form of its clinical phenotype, is largely assumed to be a multigenic disorder. Thus, neither WHSCR nor WHSCR-2 was established as definite genetic cause of WHS, but they allowed further exploration of possible candidate genes. De novo variation in *NSD2* (also known as *WHSC1*) is thought to be related to diseases since identified in patients with a wide range of phenotypic features including developmental delay, autism, and congenital cardiac disorders. Recent reported cases with de novo truncating variants on *NSD2* and detail phenotypic features have offered a new insight into genetic causes of WHS [7–9]. *NSD2* is thought to be the major, but not the unique gene for facial dysmorphisms, growth delay and intellectual disability. Here we report a boy with de novo variant in *NSD2* with atypical WHS clinical manifestations, further supported the pathogenesis of truncating variant in *NSD2* and delineated the possible symptom spectrum caused by these variants on a single gene.

## Case presentation

**Fig. 1:**
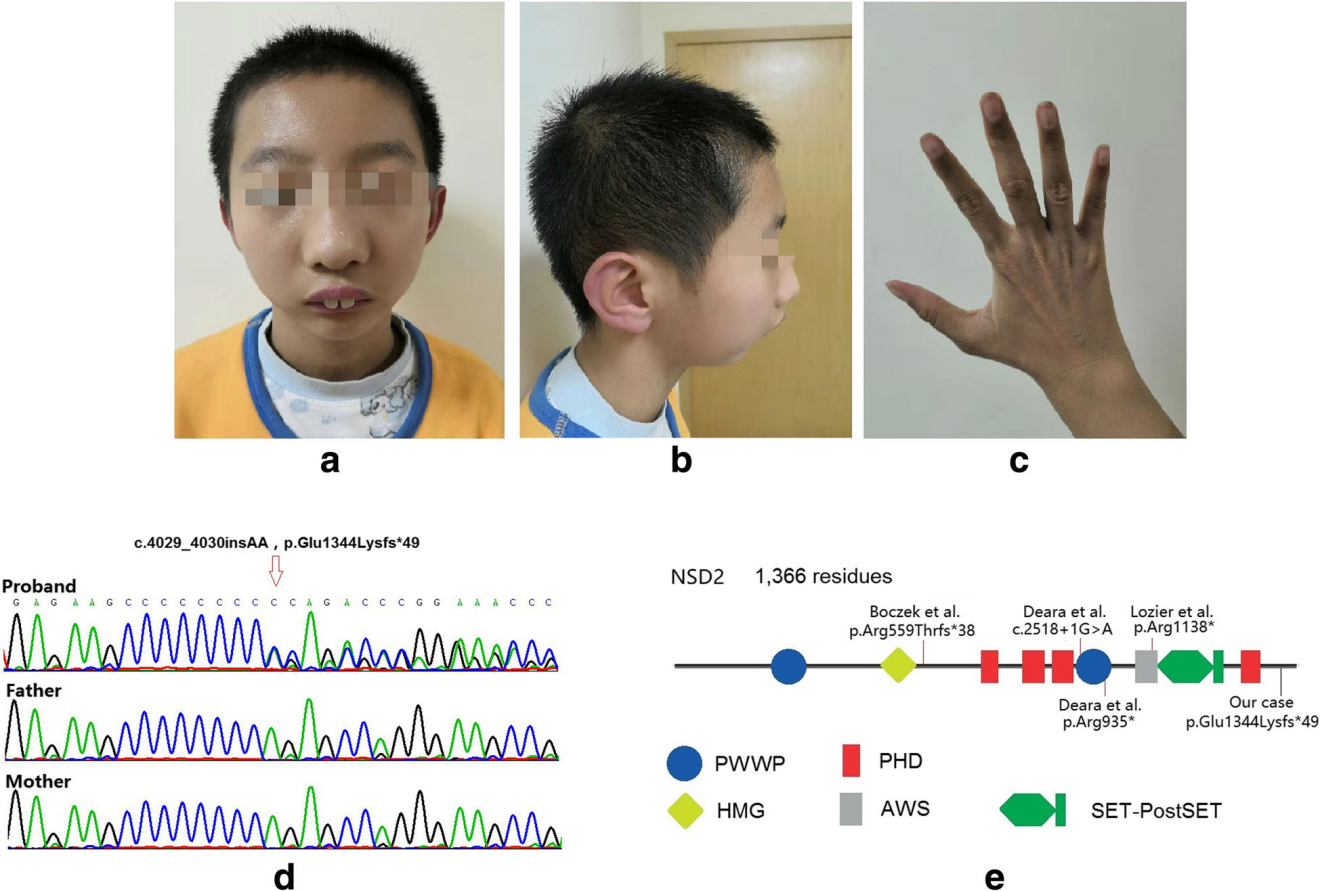
**a** & **b** Facial features of the proband including wide-spaced eyes, prominent nasal bridge continuing to forehead, abnormal teething and micrognathia. **c** Mild clinodactyly was noticed on both hands. **d** Sanger confirmation of the truncating variants detected in the pedigree. **e** Sketch map of *NSD2* gene domains and variants location of previously reported cases. (PWWP: Pro-Trp-Trp-Pro conserved motif; HMG: High mobility group box; PHD: Plant Homeodomain finger; AWS: associated with SET domain; SET: suppressor of variegation, enhancer of zeste, and Trithorax domain)

The patient, a 12-year-old boy, the only child of healthy unrelated parents, was referred to the department of developmental behavior of Shanghai Children’s Medical Center due to delayed development and growth. He was born with a cesarean section at term with a birth weight of 2.2 kg but without been told had intrauterine growth retardation during pregnancy period. The boy was purple after birth and improved after stimulation, but had feeding difficulties, so the boy was subsequent admission to the neonatal intensive care unit for weight management. Feeding difficulties and developmental delay were observed in infancy. He only sat at 10 months, walked at 18 months and spoke his first sentences at 2 years old. Growth delay persisted (current weight is 31.5 kg [P3–10], height 146.8 cm [P10–25], body mass index 14.62 kg/m2 [P3–5], and occipitofrontal circumference 52 cm [− 0.14SD] according to Chinese domestic data. Mild clinodactyly of both hands and abnormal facial features including wide-spaced eyes, prominent nasal bridge continuing to forehead, abnormal teething and micrognathia were noticed (Fig. 1 & Table 1). There was no history of seizures. Brain magnetic resonance image (MRI), EEG, X-ray of chest and spine, echocardiography and abdominal ultrasound were normal. Molecular karyotyping was normal.

**Table 1:** Clinical manifestation comparison in patients reported with truncating variants in NSD2

	WHSC1 variant	Facial Features												Prenatal onset growth deficiency	Developmental delay/intellectual disability	Seizure disorder	Feeding difficulties	Hypotonia and muscle underdevelopment	Birthweight
		“Greek warrior helmet” appearance of the nose	Microcephaly	High anterior hairline with prominent glabella	Craniofacial asymmetry	Abnormal teething	Widely spaced eyes	Epicanthus	Highly arched eyebrows	Short philtrum	Downturned corners of the mouth	Micrognathia	Poorly formed ears with pits/tags						
Patient 1^a^	c.2518 + 1G > A	Yes	Yes	No	Yes (mild)	Yes	Yes (mild)	No	Yes (mild)	Yes	Yes	Yes	Yes	Yes	Yes	No	Yes	Yes	1.5 kg[<P3]
Patient 2^a^	c.2803C > T p.Arg935*	Yes (mild)	Yes	No	Yes	Yes	No	No	No	Yes	Yes	Yes (mild)	Yes	Yes	Yes	No	Yes	Yes	2.3 kg[P3]
Patient 3^b^	c.3412C > T p.Arg1138*	No	Yes	Yes	No	Yes	Yes	Yes	No	No	No	No	Yes	No	Yes	No	No	Yes	3.05 kg[P20]
Patient 4^c^	c.1676_1679del p.Arg559Thrfs*38	No	No	No	No	No	Yes	Yes	Yes	No	No	Yes (mild)	Yes	No	Yes	No	Yes	Yes (mild)	2.58 kg[P6]
Our case	c.4029_4030insAA p.Glu1344Lysfs*49	Yes	No	Yes	No	Yes	Yes	Yes	Yes (mild)	Yes	Yes	Yes	No	Yes	Yes	No	Yes	Yes	2.2 kg[P2]

^a^ Deara et al.; ^b^ Lozier et al.; ^c^ Boczek et al.

Ethical approval for this study was obtained from the ethics committee of Shanghai Children’s Medical Center, Shanghai Jiaotong University School of Medicine. Written informed consent permit to pusblish was obtained from the proband’s parents.

Whole exome capture was performed with Agilent SureSelect V6 enrichment capture kit (Agilent Technologies, Inc., Woburn, MA, U.S.) according to the manufacturer’s instructions. The captured library was then sequenced on an Illumina HiSeq 2500 System (Illumina, Inc., San Diego, CA, U.S.). Raw sequencing data was process as previously described [10]. All detected variants were analyzed on the TGex (Translational Genomics Expert) platform featuring with the VarElect scoring system [11]. An insertion variant c.4029_4030insAA leading to frameshift mutation (p.Glu1344Lysfs*49) on NSD2 was detected and thought to have high probability as a candidate mutation.

The primers for amplification of the *NSD2* gene (NM_ 133,330.2) were designed using UCSC Exon Primer online software (http://genome.ucsc.edu/index.html) and synthesized. The primer sequences for the truncating variant to be confirmed were forward5’-agtttgtctgcccgtcctgt-3′ and reverse 5′-TGAGGATGGCTCAGTGGTG-3′.

The target fragments were amplified from the patients as well as his parents using polymerase chain reaction (Takara Biotechnology, Co., Ltd., Dalian, China). The PCR products were sequenced using an ABI3730XL sequencer (Applied Biosystems; Thermo Fisher Scientific, Inc., Waltham, MA, U.S.) with the forward and reverse primers. The sequence data were analyzed using Mutation Surveyor® software Version 4.0.4 (SoftGenetics, LLC). The truncating variant has been confirmed as de novo variants in the proband.

## Discussion and conclusion

Critical pathogenic regions responsible for microdeletion syndromes are mapped by overlapping deletions and could in some cases help to reveal a single causative gene for the syndrome. For patients with WHS, *NSD2* is the possible pathogenic gene located in the overlapping critical region previously defined by clinical and genomic information [12]. *NSD2*, nuclear receptor-binding SET domain-protein 2 (also known as WHSC1, Wolf-Hirschhorn syndrome candidate 1), is a SET domain histone methyltransferase, responsible for the methylation of HEK36. The importance of histone modification in brain development was gradually recognized as many pathogenic allele in genes encoding histone modification components was identified [13]. The histone substrate specificity of *NSD2* encoded protein may explain differences in clinical phenotypes in related patients [14]. *NSD2* gene is thought to be intolerant of loss of function variants (pLi = 1.00). Sequencing data from large cohorts of developmental delay, autism, and congenial cardiac problems also revealed several de novo variants in *NSD2* that may be related to disease [15]. Case report with de novo truncating mutations on *NSD2* with very detailed clinical information further supported that loss-of-function variants in *NSD2* likely contributes to atypical or part of the clinical spectrum of WHS [7–9]. Herein, we reported another patient with disease-related frameshift mutation on *NSD2* and reviewed clinical manifestation of previously reported patients.

Minimal diagnostic criteria for WHS have been proposed by including typical facial appearance, mental retardation, congenital hypotonia, growth delay and seizures [6]. The “Greek warrior helmet” appearance of the nose (wide bridge of the nose continuing to the forehead) is the most recognizable facial features for patients with WHS, but might become less evident after puberty. Our patient was considered for genetic testing initially due to observation of obvious abnormal appearance of the nose. Reviewing four reported cases and our case, their facial features are less recognizable comparing to typical 4p16.3 deletion WHS patients. Developmental delay or intellectual disability was observed in all four detailed reported cases. Battaglia et al. found that the degree of intellectual disability varies in patients with typical 4p deletion caused WHS [2]. The severity of intellectual disability seems to be much milder in reviewed and our patient. Seizures occur in 90–100% of children with WHS with a peak onset incidence around six to 12 months and represent one of the greatest problems in the clinical management of WHS and they act as an independent prognostic factor for the final degree of intellectual disability. Interestingly, none of the five patients reviewed with de novo truncating *NSD2* variants had epileptic symptoms, indicating that other genes located within 4p16.3 region might be responsible for seizure pathogenesis. *LETM1* and additional genes residing on 4pter distally to it have been suggested as candidate genes for seizure disorders in WHS patients in a comorbidity model of pathogenesis [16]. Thus, patients with truncating variants in *NSD2* and without seizure attack before the age of one might have better intellectual performance. Skeletal anomalies were observed in our patients. He has mild clinodactyly on both hands. The 16 month-old boy reported by Lozier et al. also manifest clinodactyly of fifth fingers of the left hand [9]. Hand malformation is a much milder symptom compared to other skeletal anomalies like kyphosis/scoliosis with malformed vertebral bodies, accessory or fused ribs, clubfeet, and split hand found in 60–70% of WHS patients. Thus, we believe that truncating variants on *NSD2* gene will lead to atypical clinical manifestations of WHS. Those patients may not have typical facial features and epileptic symptoms. The proposed minimal diagnostic criteria may not be suitable for these patients, and the optimal diagnosis criteria for them should including identification of null variants on *NSD2* gene by genetic testing.

In conclusion, we identified a de novo truncating variant on *NSD2* gene in a patient with mild phenotypic spectrum of WHS, further supported the pathogenesis of null variants in *NSD2* in syndromic intellectual disability and developmental delay and these variants lead to a mild form of WHS.

## Data Availability

The datasets (whole-exome sequencing and Sanger sequencing files) used and/or analysed during the current study are available from the corresponding author on reasonable request.

## Abbreviations

NSD2: Nuclear receptor-binding SET domain-protein 2
pLi: Probability of loss-of function variants intolerance
WHS: Wolf-Hirschhorn syndrome
WHSCR: WHS critical region
